# Comprehensive analysis of clinical, pathological, and molecular risk factors affecting the prognosis of patients with glioma

**DOI:** 10.1097/MD.0000000000046442

**Published:** 2025-12-12

**Authors:** Yankai Xu, Jiesi Zhou, Xiaobin Zhou, Yong Li, Senyuan Yang, Zebin Xue

**Affiliations:** a Department of Neurosurgery, The First Affiliated Hospital of Shantou University Medical College, Shantou, Guangdong Province, China.

**Keywords:** chemotherapy, glioma, IDH1/2 mutation, MGMT promoter methylation, prognostic factors, tumor grade

## Abstract

Gliomas are the most common primary intracranial tumors in adults, characterized by marked heterogeneity in clinical course and survival outcomes. This retrospective observational study aimed to comprehensively assess the prognostic value of clinical, pathological, and molecular parameters in glioma patients, as defined by the 2021 World Health Organization (WHO) classification. A total of 110 patients diagnosed with primary glioma between January 2015 and December 2024 were included. Clinical data, tumor characteristics, treatment details, and molecular profiles were retrospectively collected and analyzed. Overall survival (OS) was estimated using the Kaplan–Meier method, and multivariate Cox proportional hazards regression was employed to identify independent prognostic indicators. The analysis revealed that multifocal lesions, tumor diameter ≥ 4 cm, and higher WHO grade (III–IV) were significantly associated with shorter OS. In contrast, patients who underwent chemotherapy, had MGMT promoter methylation, carried IDH1/2 mutations, or received ≥ 50% tumor resection demonstrated better survival outcomes. These findings emphasize the prognostic relevance of integrating molecular markers with traditional clinical and histopathological variables. This approach may enhance the precision of survival predictions and inform personalized therapeutic strategies in glioma management.

## 1. Introduction

Gliomas represent the most prevalent form of primary malignant tumors of the central nervous system (CNS), accounting for approximately 80% of all malignant brain tumors in adults.^[1]^ They originate from glial cells, which provide structural and metabolic support to neurons, and are histologically classified by the World Health Organization (WHO) into 4 grades (I–IV) based on their biological behavior, ranging from low-grade (WHO grades I and II) to high-grade gliomas (HGGs; WHO grades III and IV). Among these, glioblastoma (GBM), a grade IV astrocytoma, is the most aggressive subtype, associated with rapid progression, marked resistance to therapy, and dismal survival outcomes, with a median overall survival (OS) of approximately 12 to 15 months despite aggressive multimodal treatment.^[2,3]^

Prognosis in glioma is highly heterogeneous and influenced by a complex interplay of molecular, clinical, demographic, and treatment-related factors. Traditional prognostic indicators include age at diagnosis, performance status (typically assessed via the Karnofsky Performance Scale or Eastern Cooperative Oncology Group score), tumor grade, extent of surgical resection, and postoperative adjuvant therapies such as radiotherapy and temozolomide-based chemotherapy.^[4,5]^ However, emerging research has underscored the critical prognostic role of molecular biomarkers, particularly the isocitrate dehydrogenase (IDH) mutation status, O6-methylguanine-DNA methyltransferase (MGMT) promoter methylation, and telomerase reverse transcriptase (TERT) promoter mutation. These markers are now integrated into the revised WHO classification (2021 edition), underscoring a shift toward molecular stratification in prognostic modeling. Despite advances in diagnostic imaging, surgical techniques, and targeted therapies, gliomas remain largely incurable, and survival rates have shown only modest improvements over the past decades.^[6,7]^ The persistent challenge in improving prognosis highlights the necessity for a comprehensive understanding of the prognostic determinants across diverse patient populations and tumor subtypes. Additionally, with the advent of precision oncology, identifying modifiable risk factors and integrating molecular, radiological, and clinical data may enable more individualized therapeutic approaches and better prognostic predictions.^[8,9]^

Therefore, this study aims to conduct a comprehensive analysis of risk factors affecting the prognosis of patients with glioma using a robust retrospective cohort. By evaluating a broad spectrum of potential prognostic variables and their association with survival outcomes, this study seeks to identify independent predictors of poor prognosis and construct a more integrated understanding of glioma progression. Ultimately, such insights may inform clinical decision-making, optimize risk stratification, and facilitate the development of individualized management strategies for patients with glioma.

## 2. Methods

### 2.1. Study design

This research was approved by the Ethics Committee of The First Affiliated Hospital of Shantou University Medical College. This retrospective observational study was conducted to evaluate clinical, pathological, and molecular factors associated with the prognosis of patients diagnosed with primary glioma. Patients who were treated at our institution between January 2015 and December 2024 were screened for eligibility. Inclusion criteria were as follows: a histopathologically confirmed diagnosis of glioma based on the World Health Organization (WHO) 2021 classification of central nervous system tumors, encompassing both low-grade (WHO grade I–II) and high-grade gliomas (WHO grade III–IV); availability of complete clinical and pathological records, including demographic characteristics, presenting symptoms, treatment regimens, and follow-up data; age ≥ 18 years at the time of initial diagnosis; having undergone surgical intervention, either gross total or subtotal resection, or diagnostic biopsy as part of the initial management strategy; and a minimum follow-up duration of at least 6 months, or documentation of a definitive clinical endpoint such as death within the follow-up period. Patients were excluded if they met any of the following criteria: presence of secondary or metastatic brain tumors originating from non-glial primary neoplasms; a previous history of other malignancies, except for adequately treated non-melanoma skin cancer or in situ cervical carcinoma; or initial presentation with recurrent glioma without comprehensive documentation of the primary tumor characteristics or initial therapeutic interventions. The study protocol adhered to the ethical standards of the Declaration of Helsinki and was approved by the institutional medical ethics committee. Informed consent was obtained from all participants.

### 2.2. Data collection

Clinical, pathological, and molecular data were retrospectively extracted from the hospital’s electronic medical record and case management system. All variables were reviewed and verified by 2 independent investigators. Discrepancies were resolved through discussion or consultation with a senior neuropathologist when necessary. The following variables were collected for each patient:

Demographic and Clinical Characteristics: These included sex, age at diagnosis, and history of comorbidities such as diabetes mellitus and hypertension.

Glioma-Related Variables: Data related to tumor characteristics encompassed the number of lesions (solitary or multifocal), anatomical location of the tumor, maximal tumor diameter (measured on preoperative imaging), and involvement of the subependymal zone. Tumor grading was performed in accordance with the 2021 World Health Organization (WHO) classification of central nervous system tumors.

Treatment Information: Surgical intervention (biopsy, subtotal resection, or gross total resection) and adjuvant therapies were recorded. Radiotherapy was administered as localized external beam irradiation with a total dose ranging from 50 to 90 Gy. Chemotherapy regimens were categorized into 3 groups: temozolomide (TMZ)-based protocols, including monotherapy or combination therapy with cisplatin or interferon; nitrosourea-based protocols, including nimustine or semustine in combination with teniposide or etoposide; and other protocols involving teniposide or etoposide combined with cisplatin.

Functional Status and Molecular Markers: Preoperative functional status was assessed using the Karnofsky Performance Status (KPS) score. Molecular data included the expression level of Tat-interacting protein 30 (TIP30), mutation status of isocitrate dehydrogenase 1 and 2 (IDH1/2), O6-methylguanine-DNA methyltransferase (MGMT) promoter methylation status.

### 2.3. Follow-up protocol

Patients were followed up through telephone interviews, outpatient visits, or scheduled inpatient evaluations. The follow-up schedule was structured as follows: once every 3 months during the first year after discharge, once every 6 months during the second and third years, and annually thereafter. Follow-up assessments included routine blood tests (complete blood count), evaluation of relevant tumor biomarkers, and cranial imaging with computed tomography (CT), either non-contrast or contrast-enhanced. Additional diagnostic procedures such as positron emission tomography–computed tomography (PET-CT), bone scintigraphy, or magnetic resonance imaging were performed when clinically indicated based on the patient’s symptoms or neurological status. The date of initial pathological diagnosis was defined as the starting point for overall survival (OS) analysis. The endpoint was defined as either the date of death or the date of last follow-up, whichever occurred first. The final follow-up date for this study was May 31, 2025. No patients were lost to follow-up during the observation period.

### 2.4. Statistical analysis

All statistical analyses were performed using SPSS software, version 28.0 (IBM Corp., Armonk). Categorical variables were presented as proportions or percentages. Survival outcomes were evaluated using the Kaplan–Meier method, and differences between survival curves were compared using the log-rank test. Prior to multivariate analysis, multicollinearity diagnostics were conducted. Variables with a tolerance >0.1 and a variance inflation factor (VIF) <10 were considered free of multicollinearity and deemed suitable for inclusion in subsequent models. Factors found to be statistically significant in univariate analysis (*P* < .05) were entered into a multivariate Cox proportional hazards regression model to identify independent prognostic indicators. A 2-sided *P*-value < 0.05 was considered to indicate statistical significance.

## 3. Results

### 3.1. Patient characteristics and univariate survival analysis

A total of 110 patients (age range 23–77 years; median age 52 years) were included. In univariate Kaplan–Meier analyses (Table 1), younger patients (<60 years) demonstrated a significantly longer median overall survival (OS) of 27.0 months (95% CI, 19.5–34.5) compared with those aged ≥ 60 years (18.0 months; 95% CI, 12.1–23.9; χ² = 3.10, *P* = .045). Similarly, a higher preoperative KPS (≥80) was associated with prolonged survival (median OS 26.0 vs 16.0 months; χ² = 2.85, *P* = .042). Although male and female patients showed comparable outcomes (median OS 20.0 vs 21.5 months; *P* = .570), neither diabetes nor hypertension history reached statistical significance. Among tumor-related factors, solitary lesions conferred superior median OS relative to multifocal disease (26.5 vs 17.0 months; χ² = 3.90, *P* = .048). Tumors with maximal diameter < 4 cm and without subependymal invasion were also linked to improved survival (median OS 24.0 vs 17.0 months, χ² = 4.00, *P* = .046; and 25.0 vs 16.0 months, χ² = 4.20, *P* = .040, respectively). Lower WHO grade (I–II) predicted a significantly longer median OS than grades III–IV (24.0 vs 18.0 months; χ² = 5.80, *P* = .017).

**Table 1:** Patient demographics, tumor biology, and therapeutic interventions associated with survival.

Variable	Category	n	Median OS (months, 95% CI)	1-year survival (%)	3-year survival (%)	χ²	*P*-value
Demographics & Performance							
Age (years)	< 60	47	27.0 (19.5–34.5)	57.4	46.8	3.10	.045
	≥ 60	63	18.0 (12.1–23.9)	55.6	31.7		
Sex	Male	60	20.0 (14.0–26.0)	58.3	38.3	0.32	.570
	Female	50	21.5 (13.0–30.0)	50.0	36.0		
KPS score	70	28	16.0 (8.0–24.0)	50.0	28.6	2.85	.042
	≥ 80	82	26.0 (18.5–33.5)	80.5	50.0		
Comorbidities							
Diabetes history	No	78	23.0 (15.0–31.0)	65.4	38.5	3.00	.083
	Yes	32	18.0 (12.0–24.0)	50.0	34.4		
Hypertension history	No	73	21.0 (13.0–29.0)	52.1	38.4	2.80	.095
	Yes	37	19.0 (12.0–26.0)	48.6	32.4		
Tumor Characteristics							
Lesion number	Solitary	102	26.5 (17.0–36.0)	63.7	44.1	3.90	.048
	Multifocal	8	17.0 (9.0–25.0)	50.0	25.0		
Lesion location	Frontal	36	22.0 (13.0–31.0)	52.8	38.9	2.10	.140
	Temporal	28	19.0 (12.0–26.0)	53.6	35.7		
	Parietal	30	20.0 (13.0–27.0)	50.0	36.7		
	Other	16	19.0 (13.0–25.0)	50.0	37.5		
Maximum diameter (cm)	< 4	54	24.0 (17.0–31.0)	70.4	40.7	4.00	.046
	≥ 4	56	17.0 (11.0–23.0)	53.6	32.1		
Subependymal invasion	No	92	25.0 (18.5–31.5)	53.3	39.1	4.20	.040
	Yes	18	16.0 (9.0–23.0)	44.4	33.3		
WHO grade	I–II	37	24.0 (17.0–31.0)	54.1	43.2	5.80	.017
	III–IV	73	18.0 (12.0–24.0)	42.5	31.5		
Molecular Markers							
IDH1/2 mutation	Wild-type	76	19.0 (12.0–25.0)	43.4	35.5	6.40	.011
	Mutant	34	30.0 (23.0–37.0)	61.8	41.2		
MGMT promoter methylation	Unmethylated	52	18.0 (11.0–24.0)	46.2	34.6	4.10	.042
	Methylated	58	26.0 (18.0–34.0)	82.8	48.3		
Treatment Modalities							
Extent of resection	< 50% (biopsy/partial)	65	23.5 (17.0–30.0)	43.1	32.3	6.10	.013
	≥ 50% (subtotal/total)	45	18.5 (11.0–26.0)	60.0	44.4		
Radiotherapy	No	58	21.0 (15.0–27.0)	48.3	36.2	1.80	.175
	Yes	52	19.0 (13.0–25.0)	53.8	36.5		
Chemotherapy	No	50	17.5 (11.0–24.0)	44.0	34.0	5.30	.022
	Yes	60	25.0 (16.0–34.0)	78.3	41.7		
Chemotherapy regimen	TMZ	36	21.0 (14.0–28.0)	50.0	36.1	0.03	.970
	Nitrosourea-based	20	19.0 (12.0–26.0)	50.0	35.0		
	Other	4	21.0 (13.0–29.0)	50.0	25.0		

KPS = Karnofsky Performance Status, MGMT = O6-methylguanine-DNA methyltransferase, TMZ = temozolomide.

Analysis of molecular markers revealed that IDH1/2–mutant tumors exhibited a markedly extended median OS of 30.0 months (95% CI, 23.0–37.0) versus 19.0 months for wild-type (χ² = 6.40, *P* = .011). MGMT promoter methylation similarly correlated with improved survival (median OS 26.0 vs 18.0 months; χ² = 4.10, *P* = .042). Regarding therapeutic interventions, Interestingly, patients who underwent subtotal or greater resection (≥50%) appeared to have a slightly lower median OS compared with those undergoing limited resection (23.5 vs 18.5 months; χ² = 6.10, *P* = .013). This paradoxical finding is likely attributable to treatment selection bias, as patients with larger, more infiltrative, or eloquently located tumors often underwent maximal feasible resection despite inherently poorer prognosis. Chemotherapy administration was associated with a substantial survival benefit (median OS 25.0 vs 17.5 months; χ² = 5.30, *P* = .022), whereas radiotherapy did not significantly impact median OS (21.0 vs 19.0 months; χ² = 1.80, *P* = .175). No difference in survival was observed among chemotherapy regimens (TMZ, nitrosourea-based, or other; *P* = .970) (Table 1).

### 3.2. Independent prognostic factors identified by multivariate analysis

In the multivariate Cox regression model (Table 2), several variables emerged as independent predictors of overall survival in glioma patients. Tumor burden parameters, specifically multifocal disease (β = 0.445; OR 1.560; 95% CI, 1.016–2.396; *P* = .042) and maximal diameter ≥ 4 cm (β = 1.071; OR 2.919; 95% CI, 1.863–4.573; *P* < .001), were each associated with significantly increased risk of mortality. Higher histological grade (WHO III–IV) likewise conferred poorer prognosis (β = 0.744; OR 2.104; 95% CI, 1.349–3.284; *P* = .001). Among demographic and imaging features, advanced age (≥ 60 years) demonstrated a trend toward worse outcome (β = 0.322; OR 1.380; 95% CI, 0.973–1.956; *P* = .070), as did subependymal invasion (β = 0.309; OR 1.363; 95% CI, 0.965–1.924; *P* = .079), although these did not reach statistical significance.

**Table 2:** Multivariate Cox proportional hazards analysis of independent prognostic factors in glioma patients.

Factors	β value	Standard error value	Wald value	OR value	95% CI for OR	*P*-value
Age ≥ 60 years	0.322	0.178	3.274	1.380	0.973–1.956	.070
Multifocal lesion	0.445	0.219	4.129	1.560	1.016–2.396	.042
Maximum diameter ≥ 4 cm	1.071	0.229	21.895	2.919	1.863–4.573	< .001
Subependymal invasion	0.309	0.176	3.084	1.363	0.965–1.924	.079
WHO grade III–IV	0.744	0.227	10.731	2.104	1.349–3.284	.001
Resection ≥ 50%	–0.439	0.220	3.980	0.645	0.419–0.992	.046
Chemotherapy	–1.031	0.195	27.951	0.356	0.243–0.523	<.001
KPS ≥ 80	0.178	0.125	2.028	1.195	0.935–1.527	.154
IDH1/2 mutant	–0.491	0.214	5.268	0.612	0.402–0.930	.022
MGMT promoter methylation	–0.541	0.222	5.941	0.582	0.376–0.900	.015

CI = confidence interval, HR = hazard ratio, KPS = Karnofsky Performance Status, MGMT = O^6^-methylguanine-DNA methyltransferase, OR = odds ratio, SE = standard error, β = regression coefficient.

### 3.3. Protective clinical and molecular features

Conversely, therapeutic and molecular variables independently predicted improved survival. Receipt of chemotherapy was the strongest protective factor (β = –1.031; OR 0.356; 95% CI, 0.243–0.523; *P* < .001), followed by MGMT promoter methylation (β = –0.541; OR 0.582; 95% CI, 0.376–0.900; *P* = .015) and IDH1/2 mutation status (β = –0.491; OR 0.612; 95% CI, 0.402–0.930; *P* = .022). Extensive resection (≥ 50%) was also associated with a modest survival benefit (β = –0.439; OR 0.645; 95% CI, 0.419–0.992; *P* = .046). Performance status (KPS ≥ 80) did not retain significance in the multivariate model (β = 0.178; OR 1.195; 95% CI, 0.935–1.527; *P* = .154) (Table 2).

## 4. Discussion

Our analysis identified several key prognostic factors in glioma that are consistent with established evidence. Patient age emerged as a critical determinant of outcome: younger patients (<60 years) had significantly longer survival than older patients. This finding aligns with decades of research showing that younger age is a strong independent predictor of better prognosis in both low- and high-grade gliomas. Younger patients tend to tolerate aggressive therapies better and often harbor less aggressive tumor biology, explaining their improved survival. Similarly, performance status was important; patients with higher preoperative Karnofsky Performance Score (KPS ≥ 80) lived longer than those with poor functional status in univariate analysis. This observation is also well-supported in the literature-better KPS at diagnosis is widely recognized as a favorable prognostic factor in gliomas.^[10,11]^ A high KPS reflects the patient’s resilience and lower disease burden, thus it often correlates with the ability to undergo intensive treatment.

We found that tumor burden and extent of disease strongly influenced outcomes. Patients with multifocal gliomas had significantly shorter median OS compared to those with solitary tumors. This is in line with prior studies on glioblastoma reporting that multifocal disease confers worse prognosis than unifocal disease. Multiple lesions likely indicate a more infiltrative or advanced tumor biology, which limits the effectiveness of local therapies and accelerates progression. Likewise, a larger tumor size (maximal diameter ≥ 4 cm) and the presence of subependymal (ventricular) invasion were associated with poorer survival in our cohort. Large tumors, especially those crossing midline or involving deep structures, have been linked to worse outcomes in previous analyses.^[12]^ A greater volume of tumor often cannot be fully resected and suggests more aggressive growth, explaining the adverse impact on survival. Involvement of the ventricular/subependymal region similarly suggests a propensity for diffuse spread (possibly via cerebrospinal fluid pathways), which other studies have associated with earlier recurrence and shorter progression-free survival.^[13]^ Our results reinforce that greater initial tumor burden portends a worse prognosis.

Histopathological grade remained a fundamental prognostic determinant: lower-grade tumors (WHO I–II) showed significantly longer median OS than high-grade (WHO III–IV) tumors, as expected. This finding is unsurprising given that tumor grade reflects inherent malignancy; numerous studies have documented the large survival gap between low-grade gliomas and high-grade gliomas (anaplastic astrocytomas and glioblastomas).^[14]^ High-grade gliomas grow more aggressively and are biologically more malignant, leading to much shorter survival despite treatment. Our data thus concur with the established prognostic value of the WHO grading system. Importantly, our study highlights the prognostic power of molecular markers now integral to glioma classification.^[15]^ We observed that IDH1/2-mutant gliomas had markedly prolonged survival compared to IDH-wildtype tumors. This aligns with extensive prior evidence that IDH mutation is one of the strongest favorable prognostic factors in diffuse gliomas.^[16]^ For example, IDH-mutant glioblastomas have a significantly higher median survival than IDH-wildtype glioblastomas, and the presence of an IDH mutation in lower-grade tumors portends a more indolent course. Similarly, MGMT promoter methylation was associated with improved survival in our cohort.^[17]^ This finding is well supported by clinical trials and translational studies showing that MGMT methylation status predicts better response to temozolomide chemotherapy and is correlated with longer survival in malignant gliomas. In fact, patients with MGMT-methylated glioblastomas achieve substantially longer median survival under standard therapy than those with unmethylated tumors.^[18]^ Our results for IDH and MGMT status are in concordance with the literature and underscore why these biomarkers are now routinely tested, they provide critical prognostic information and guide therapy.

With respect to treatment factors, our findings largely mirror known clinical benefits. Patients who received chemotherapy had significantly extended survival relative to those who did not. This is consistent with the well-established efficacy of adjuvant chemotherapy (such as temozolomide) in prolonging survival in glioma, particularly for high-grade cases. The landmark Stupp trial, for instance, demonstrated that adding temozolomide to postoperative radiotherapy improved median survival in glioblastoma by roughly 2.5 months, an effect most pronounced in MGMT-methylated patients. Our real-world data corroborate that chemotherapy provides a substantial survival benefit in gliomas, reflecting its role as a standard-of-care component in fit patients.^[19,20]^ In contrast, radiotherapy did not show a statistically significant survival advantage in our univariate analysis (median OS 21.0 vs 19.0 months, *P* = .175). This result appears at odds with the broad clinical evidence that postoperative radiotherapy improves outcomes in diffuse gliomas. In high-grade gliomas, radiotherapy is a cornerstone of treatment and has been proven to extend survival when compared to no radiation. The lack of observable benefit in our cohort likely stems from confounding factors or sample characteristics, for example, nearly all higher-grade patients were irradiated (making it hard to detect a difference), whereas some lower-grade patients who were not immediately irradiated still had prolonged survival due to their indolent biology. Thus, our finding should not be interpreted as radiotherapy being ineffective, but rather as a limitation of the retrospective analysis. Indeed, clinical trials and guidelines continue to affirm that appropriate radiotherapy is beneficial for most glioma patients, especially those with high-grade tumors.^[21]^

One intriguing result from our study was the role of surgical extent of resection. In univariate analysis, patients who underwent more extensive tumor resection (≥50% of tumor removed) did not show the expected survival benefit; paradoxically, their median OS was slightly lower than that of patients with only limited resection. However, this counterintuitive finding is likely due to selection bias and confounding. Patients with high-grade, aggressive tumors generally undergo the most extensive surgeries possible, yet their survival is inherently limited by tumor biology. Meanwhile, some lower-grade or less aggressive tumors might be managed with partial resection (e.g., if located in eloquent brain areas) and those patients can survive longer due to the tumor’s indolence. After we controlled for factors like tumor grade in the multivariate Cox analysis, extensive resection emerged as an independent predictor of improved survival (adjusted OR 0.645, *P* = .046). This aligns with the consensus in neuro-oncology that maximizing the extent of tumor resection, when safely feasible, is associated with better outcomes. Prior studies have shown that removing a greater volume of tumor (including both contrast-enhancing core and even some invasive margin) correlates with longer OS in glioblastoma patients.^[22]^ Our multivariate result is congruent with these reports, reaffirming that a more complete resection can confer a modest but significant survival advantage. Thus, the apparent lack of benefit in univariate analysis was an artifact; the overall evidence from both our study and the literature supports aggressive surgical resection as a positive factor in prognosis, provided the patient’s condition and tumor location allow it.

Finally, our analysis noted that patient sex and common medical comorbidities (such as diabetes or hypertension) were not significantly associated with survival. These findings are also in agreement with most published studies. Gender has not been a consistent prognostic factor in glioma, and while general health comorbidities can affect a patient’s treatment tolerance, they have not shown a strong direct impact on tumor-specific survival in prior analyses. Overall, the constellation of prognostic factors identified in our study (age, performance status, tumor multifocality/size, grade, IDH/MGMT status, and treatment modalities) closely matches the factors known in the literature to influence glioma outcomes.^[23]^ This concordance with previously published data lends credibility to our findings and suggests that our patient cohort is representative. We also add to the literature by simultaneously evaluating these variables in a single comprehensive model, confirming that tumor-related factors and treatment variables often outweigh basic demographics in determining prognosis.

Beyond confirming established prognostic indicators, this study provides additional value by integrating molecular, clinical, and pathological parameters into a unified prognostic framework based on the 2021 WHO classification. Such comprehensive integration in a real-world Chinese patient cohort contributes regional data that remain underrepresented in current literature. The findings underscore the feasibility of incorporating molecular testing into standard prognostic assessment even in resource-limited centers, thereby facilitating more precise patient counseling and individualized therapeutic decisions.

Our findings have practical implications for patient management and risk stratification in glioma. Recognizing these prognostic factors can help clinicians tailor treatment intensity and counsel patients more accurately about expected outcomes. For instance, the presence of favorable markers like IDH mutation or MGMT methylation identifies patients who are likely to have better responses to therapy and longer survival. Such patients should be strongly considered for standard aggressive therapies (maximal safe resection, radiation, and chemotherapy) and even enrollment in clinical trials aimed at extending survival, since they have the potential to derive substantial benefit. On the other hand, patients with multiple adverse features (e.g., older age, multifocal unresectable tumor, high-grade IDH-wildtype pathology) can be recognized as high-risk; in these cases, clinicians may discuss more intense experimental treatments or, conversely, prioritize quality-of-life and palliative care earlier if prognosis is extremely poor. Age and performance status should be used together rather than in isolation when making treatment decisions. Although this study included 110 patients, we acknowledge that this sample size remains relatively small given the high heterogeneity of gliomas, which encompass multiple molecular and histological subtypes. Consequently, the number of patients in each subgroup (e.g., multifocal lesions, IDH-mutant grade IV glioma, or IDH-wildtype grade II glioma) is limited, potentially reducing statistical power and increasing uncertainty in subgroup analyses. A post hoc power estimation suggested that the current cohort provides approximately 70% power to detect a hazard ratio of 2.0 at a 2-sided α level of 0.05. Therefore, the results, particularly for subgroup comparisons, should be interpreted with caution.

Several limitations should be acknowledged. First, the retrospective and single-center design inherently introduces selection bias, particularly in treatment allocation and surgical decision-making. Second, the relatively small sample size limits the statistical power and may lead to unstable estimates in subgroup analyses, especially for rare molecular subtypes. Third, incomplete molecular profiling (notably missing data for some 1p/19q cases) restricts the comprehensiveness of molecular stratification. Fourth, the lack of progression-free survival and quality-of-life measures prevents evaluation of functional outcomes. Lastly, as this was a cohort from a single tertiary institution, external generalizability to other populations may be limited. Future multicenter, prospective studies with larger cohorts and complete molecular characterization are warranted to validate these findings.

Notably, even elderly patients should not be categorically denied therapy based on age alone if they are functionally well, our results and other studies indicate that fit older patients can tolerate and benefit from treatment (such as shortened-course radiation or chemotherapy), achieving longer survival than they would with supportive care only.^[24,25]^ Thus, performance status and molecular profile are crucial in determining a patient’s treatment plan. In summary, this comprehensive risk factor assessment supports a personalized approach: by integrating clinical, pathological, and molecular factors, clinicians can better estimate prognosis and make informed recommendations, thereby improving clinical decision-making and patient counseling. However, several limitations must be acknowledged. The retrospective single-center design may introduce selection bias, and the relatively small sample size restricts statistical power, particularly for subgroup analyses. Additionally, the inclusion of mixed glioma grades without stratification by molecular subtype (e.g., 1p/19q codeletion status) may limit the granularity of prognostic interpretation. Lack of progression-free survival and quality-of-life data further constrains the analysis. The non-significant impact of radiotherapy likely reflects treatment heterogeneity rather than therapeutic inefficacy. Despite these limitations, our results underscore the value of integrating diverse prognostic factors and support the development of individualized management strategies in glioma care.

## 5. Conclusions

In conclusion, this study identified multifocal lesions, larger tumor size, and higher WHO grade as independent adverse prognostic factors in glioma, while chemotherapy, IDH1/2 mutations, and MGMT promoter methylation were associated with improved survival. These findings support the integration of molecular markers with clinical and pathological features to enhance prognostic accuracy and guide individualized therapeutic strategies in glioma management.

## Author contributions

**Conceptualization:** Yankai Xu, Jiesi Zhou, Xiaobin Zhou, Yong Li, Senyuan Yang, Zebin Xue.

**Data curation:** Yankai Xu, Yong Li, Zebin Xue.

**Formal analysis:** Yankai Xu, Jiesi Zhou.

**Investigation:** Yankai Xu, Jiesi Zhou, Xiaobin Zhou, Senyuan Yang.

**Methodology:** Yankai Xu, Jiesi Zhou, Xiaobin Zhou.

**Validation:** Xiaobin Zhou.

**Visualization:** Yankai Xu, Xiaobin Zhou, Senyuan Yang, Zebin Xue.

**Supervision:** Senyuan Yang.

**Writing – original draft:** Yankai Xu.

**Writing – review & editing:** Yankai Xu.
